# Factors affecting duration of SARS-Cov-2 viral shedding in mildly symptomatic patients isolated in a community facility

**DOI:** 10.1371/journal.pone.0257565

**Published:** 2021-09-27

**Authors:** Hui Mei Cheng, Xiahong Zhao, Wei Shyann Lim, Beatrice Jia Min Tan, Hong Liang Tey

**Affiliations:** 1 National Skin Centre, Singapore, Singapore; 2 Yong Loo Lin School of Medicine, National University of Singapore, Singapore, Singapore; 3 Lee Kong Chian School of Medicine, Nanyang Technological University, Singapore, Singapore; Ohio State University Wexner Medical Center Department of Surgery, UNITED STATES

## Abstract

**Introduction:**

Mildly symptomatic cases of Covid-19 in previously-well individuals form the majority of infections and also serve as potent vectors of transmission. The factors affecting the duration of SARS-CoV-2 RNA viral shedding (DVS) in these patients remain largely unknown.

**Objectives:**

To perform a systematic analysis of the clinical, radiologic, laboratory investigations in patients with few comorbidities infected with mild Covid-19 to identify factors associated with the DVS.

**Methods:**

In this retrospective cohort study, patients with mild or asymptomatic Covid-19 were included. Baseline characteristics including age, nationality, comorbidities, concomitant medications, and type of isolation arrangement in the facility (single or in pairs) were collected. Clinical features and radiologic/haematologic findings were also collected. Taking day 28 as the cut-off, 187 patients who had a negative swab result up to day 28 (no prolonged DVS) were compared to 126 patients with a persistently positive result on or after day 28 (prolonged DVS).

**Results:**

Of 964 consecutive patients included, 851 (88.3%) patients were symptomatic. 266 patients had a documented negative RT-PCR assay with a median DVS of 25 days (range: 13 to 96 days; interquartile range (IQR): 22 to 33 days). Patients isolated in pairs were associated with prolonged DVS (OR: 2.7; 95% CI: 1.7 to 4.5; p<0.0001) compared to those isolated individually. Among vital signs, only tachycardia was associated with prolonged DVS (OR: 2.6; 95% CI: 1.0 to 7.1; p = 0.03). Amongst investigations, only a raised CRP was associated with prolonged DVS (OR: 2.7; 95% CI: 1.1 to 6.8; p = 0.02).

**Conclusions:**

In young, mildly symptomatic Covid-19 patients, prolonged DVS was associated with being isolated in pairs compared to individually. In situations where a negative RT-PCR test result is required, retesting in patients who were not isolated individually, or who had baseline tachycardia or a raised CRP, may be delayed to increase the yield of a negative result.

## Introduction

Mildly symptomatic cases of Covid-19 form the majority of infections [1] and also serve as potent vectors of transmission [2]. The duration of viral RNA shedding (DVS) in these patients has important public health implications particularly with respect to isolation duration as well as retesting schedule and de-isolation protocols but studies are lacking. In Singapore, an outbreak of Covid-19 clusters in the foreign-worker population residing in worker dormitories led to the development of a large-scale community medical facility [3]. An understanding of the DVS and its determinants in largely well individuals would allow more effective allocation of testing resources to confirm clearance of virus and potentially allow for a stratified de-isolation protocol of these patients. Moreover, despite efforts at vaccination drives underway internationally, the incidence of Covid-19 infection and its associated mortality shows no clear signs of abatement at present [4]. An understanding of DVS remains important in directing preventive measures in addition to vaccination efforts.

The aim of this retrospective cohort study was to describe the characteristics and natural history of disease in healthy individuals with mild Covid-19 infections to determine the factors which affect DVS.

## Methods

Patients diagnosed with Covid-19 based on a positive nasopharyngeal swab reverse transcription-polymerase chain reaction (RT-PCR) result for SARS-CoV-2 and admitted from 10 to 17 April 2020 to a community care facility (Singapore EXPO Convention and Exhibition Centre) were included in this retrospective cohort study. As part of the routine clinical management of these patients, all patients underwent repeated nasopharyngeal swab tests at day 14 which was repeated every 3–5 days. Protocol of swabbing differed according to discharge destination. If a patient was discharged to the community, two nasopharyngeal swab PCR tests performed on separate days were required to be negative and no further swabs were performed after this. However, for patients who were transferred to a quarantine facility after a period of observation in this community care facility, negative PCR swab test was not a requirement. Nevertheless, if such patients received two separate negative PCRs, no further swabs were performed.

Baseline characteristics including age, nationality, comorbidities, concomitant medications, and type of isolation arrangement in the facility (single or in pairs) were collected. Presence and nature of symptoms, and baseline vital signs including presence of temperature (T), heart rate (HR), blood pressure (BP) were collected. Fever was defined as T ≥ 37.5⁰C, tachycardia as HR ≥ 100. Investigations including chest X-ray (CXR) findings, and the presence of abnormal blood results including anemia, leukopenia, leukocytosis, thrombocytopenia, thrombocytosis, raised inflammatory markers including C-reactive protein (CRP), ferritin, lactate dehydrogenase and transaminitis of liver enzymes were collected.

Nasopharyngeal swabs were obtained by inserting a swab into each nostril to a depth of approximately 5cm, done one side at a time. Viral nucleic acids were extracted by the laboratory using the the NucliSens EasyMAG instrument (BioMérieux, Marcy-l’Étoile, France). Presence of SARS-CoV-2 was assessed using the Agency for Science, Technology and Research (A*STAR) Fortitude Kit (Accelerate Technologies, Singapore). All tests were performed according to the manufacturer’s protocol. A positive RT-PCR test had cycle-threshold (Ct) value of ≤ 30.

All variables were summarized descriptively using counts and percentages for variables in nominal scale and mean with standard deviation and median with range for variables in interval/ratio scale. DVS was defined as the time from positive RT-PCR or symptom onset, whichever was earlier, to the date of negative RT-PCR on nasopharyngeal swab. Patients who had a negative swab result up to day 28 (no prolonged DVS) were compared to those with a persistently positive result on or after day 28 (prolonged DVS). Two sample t-test for interval/ratio variables and Fisher’s exact test for nominal variables were used to assess the difference. P-value less than 0.05 was considered statistically significant. Odds ratio (OR) was calculated for factors that were significantly associated with prolonged DVS with 95% confidence interval (CI). Statistical analyses were done using R version 3.5.3.

The study was approved by the National Healthcare Group Domain Specific Review Board (Approval ID 2020/00694) and followed the Strengthening the Reporting of Observational Studies in Epidemiology (STROBE) reporting guidelines. Requirement for informed consent was waived by the ethics committee. All data were anonymised at time of access.

## Results

Baseline characteristics (Table 1).

**Table 1:** Baseline characteristics, signs and symptoms, investigations of all patients.

Summary Statistics	All (n = 964)
**Baseline characteristics**	
**Age (years)**	
n; mean (SD)	947; 28.8 (4.2)
**Nationality**	
Bangladesh/Indian	821/945 (86.9%)
Others	46/945 (4.9%)
Unknown	78/945 (8.3%)
**Comorbidities**	
Yes	33/714 (4.6%)
No	681/714 (95.4%)
**Usual medications**	
Yes	11/712 (1.5%)
No	701/712 (98.5%)
**Single-bed cubicle**	
Yes	479 (49.6%)
No	485 (50.3%)
**Transferred to tertiary hospital**	
Yes	4/963 (0.4%)
No	959/963 (99.6%)
**Duration of Viral Shedding (days)**	
n; median (range) (IQR)	266; 25 (13, 96) (22, 33)
**No. of days from diagnosis to discharge**	
n; median (range) (IQR)	943; 34 (6, 72) (18, 36)
**Signs and symptoms**	
**Symptomatic**	
Yes	851 (88.3%)
No	113 (11.7%)
**Symptom duration (days)**	
n; median (range) (IQR)	677; 11 (0, 45) (10, 15)
**Symptom (n = 851)**	
Fever	478/789 (60.6%)
Upper respiratory tract symptoms	484/789 (61.3%)
Headache, myalgia, malaise	143/789 (18.1%)
Chest pain, dyspnea	14/789 (1.8%)
Diarrhea	5/789 (0.6%)
**Vital signs**	
Fever	65/960 (6.8%)
Tachycardia	81/961 (8.4%)
SpO2, % (n; mean [SD])	961; 99.5 (0.8)
SBP, mmHg (n; mean [SD])	961; 123.8 (11.6)
DBP, mmHg (n; mean [SD])	961; 81.6 (9.9)
**Investigations**	
**CXR findings**	
Normal	836/948 (88.2%)
Opacity or haziness	76/948 (8.0%)
Consolidation	2/948 (0.2%)
Others	34/948 (3.6%)
**Abnormal blood results (n = 352)**	
Anemia	17 (4.8%)
Leukopenia	100 (28.4%)
Leukocytosis	239 (67.9%)
Thrombocytopenia	39 (11.1%)
Thrombocytosis	2 (0.6%)
Raised CRP	118 (33.5%)
Raised ferritin	8 (2.3%)
Raised LDH	23 (6.5%)
Transaminitis	65 (18.5%)

All 964 consecutive patients admitted to the facility during the study period were included, all patients were male, with a mean age of 28.8 years old (SD: 4.2), and majority (86.9%) was of Bangladeshi (58.2%) or Indian (28.7%) ethnicity. 95.4% did not have any past medical history and 98.5% were not on any regular medications. Of 33 patients with comorbidities, 7 had hypertension, 4 had asthma, 3 had diabetes mellitus, the remaining had unrelated medical and surgical history (allergic rhinitis, eczema, gastroesophageal reflux disease). Correspondingly, regular medications patients were taking included antihypertensives (amlodipine, bisoprolol, enalapril), diabetic medications (linagliptin, metformin), and medications for asthma or gastroesophageal reflux disease (salbutamol inhaler, proton-pump inhibitors). 49.6% were isolated in a cubicle individually, the remainder were isolated in pairs. Four patients had clinical deterioration and were transferred to a tertiary hospital. None of the patients died from Covid-19 related or unrelated causes at 3 months follow up in July 2020. 266 patients had a documented negative RT-PCR result and median DVS was 25 days (range: 13 to 96 days; interquartile range (IQR): 22 to 33 days). Median DVS was 25 days (range: 13 to 96 days; IQR: 23 to 30 days) among symptomatic patients (n = 243) and 26 days (range: 13 to 79 days) among asymptomatic patients (n = 23) (p = 0.319). On day of discharge (median 34 days; range: 6 to 72 days; IQR: 18 to 36 days), 89.9% (n = 867) reported no symptoms.

### Signs and symptoms (Table 1)

851 (88.3%) patients were symptomatic at admission, median length of symptoms was 11 days (range 0 to 45 days, IQR 10 to 15 days). Reported symptoms were fever (60.6%) and upper respiratory tract symptoms (61.3%) including cough, rhinorrhea, sore throat, anosmia, ageusia. Vital signs on admission were largely normal; the most common abnormalities were tachycardia (8.4%) and fever (6.8%).

### Investigations (Table 1)

CXRs was performed on 948 patients, of which 836 (88.2%) were normal. Frank consolidation was seen in only 2 CXRs, and most abnormal CXRs reported ill-defined opacities or haziness (8.0%). Among blood investigations, leukocytosis (67.9%), raised CRP (33.5%), and leukopenia (28.4%) were the most common abnormalities.

### Factors affecting DVS (Table 2)

**Table 2:** Factors affecting duration of viral shedding.

Summary Statistics	All (n = 313)	Prolonged Duration of Viral Shedding		P-value
		No (n = 187)	Yes (n = 126)	
**Baseline characteristics**				
**Age (years)**				0.45
n; mean (SD)	308; 28.7 (3.9)	184; 28.5 (3.6)	124; 28.9 (4.3)	
**Nationality**				0.99
Bangladeshi/Indian	266/312 (85.3%)	158/186 (85.0%)	108 (85.7%)	
Others	46/945 (4.9%)	10 (5.3%)	4 (3.2%)	
Unknown	32/312 (10.3%)	18/186 (9.7%)	14 (11.1%)	
**Comorbidities**				0.93
Yes	12/233 (5.6%)	7/129 (5.4%)	6/104 (5.8%)	
No	220/233 (94.4%)	122/129 (94.6%)	98/104 (94.2%)	
**Usual medications**				
Yes	5/232 (2.2%)	1/128 (0.8%)	4/104 (3.8%)	0.18
No	227/232 (97.8%)	127/128 (99.2%)	100/104 (96.2%)	
**Single-bed cubicle**				
Yes	191 (61.0%)	132 (70.6%)	59 (46.8%)	**< 0.0001**
No	122 (39.0%)	55 (29.4%)	67 (53.2%)	
**Transferred to tertiary hospital**				
Yes	3/312 (1.0%)	3 (1.6%)	0/125 (0.0%)	0.28
No	309/312 (99.0%)	184 (98.4%)	125/125 (100.0%)	
**Signs and symptoms**				
**Symptomatic**				
Yes	285 (91.1%)	175 (93.6%)	110 (87.3%)	0.07
No	28 (8.9%)	12 (6.4%)	16 (12.7%)	
**Symptom onset (n = 285)**				
Fever	145/268 (54.1%)	84/163 (51.5%)	61/105 (58.1%)	0.72
Upper respiratory tract symptoms	178/268 (66.4%)	109/163 (66.9%)	69/105 (65.7%)	0.28
Chest pain, dyspnea	7/268 (2.6%)	3/163 (1.8%)	4/105 (3.8%)	0.45
Diarrhea	2/268 (0.7%)	0/163 (0.0%)	2/105 (1.9%)	0.15
Headache, myalgia, malaise	47/268 (17.5%)	28/163 (17.2%)	19/105 (18.1%)	1.00
**Symptom duration (days)**				**0.01**
n; median (range) [IQR]	222; 11 (3, 44) [9, 14]	136; 10 (3, 38) [8, 13]	86; 11 (6, 44) [10, 18]	
**Vital signs**				
Fever	26/312 (8.3%)	13/186 (7.0%)	13/126 (10.3%)	0.30
Tachycardia	24/312 (7.7%)	9/186 (4.8%)	15/126 (11.9%)	**0.03**
SpO2, % (n; mean [SD])	312; 99.5 (0.9)	186; 99.5 (0.9)	99.5 (0.8)	1.00
SBP, mmHg (n; mean [SD])	312; 124.2 (11.5)	186; 124.1 (11.6)	124.4 (11.4)	0.79
DBP, mmHg (n; mean [SD])	312; 80.8 (9.7)	186; 80.0 (9.2)	81.8 (10.4)	0.11
**Investigations**				
**CXR findings**				
Normal	266/306 (86.9%)	166/185 (89.7%)	100/121 (82.6%)	0.11
Opacity or haziness	27/306 (8.8%)	11/185 (5.9%)	16/121 (13.2%)	
Consolidation (Unilateral)	1/306 (0.3%)	1/185 (0.5%)	0/121 (0.0%)	
Others	12/306 (3.9%)	7/185 (3.8%)	5/121 (4.1%)	
**Abnormal blood results (n = 118)**				
Anemia	10/118 (8.5%)	8/82 (9.8%)	2/36 (5.6%)	0.72
Leukocytopenia	33/118 (28.0%)	23/82 (28.0%)	10/36 (27.8%)	1.00
Leukocytosis	79/118 (66.9%)	54/82 (65.9%)	25/36 (69.4%)	0.83
Thrombocytopenia	15/118 (12.7%)	10/82 (12.2%)	5/36 (13.9%)	0.77
Thrombocytosis	1/118 (0.8%)	1/82 (1.2%)	0/36 (0.0%)	1.00
Raised CRP	37/118 (31.4%)	20/82 (24.4%)	17/36 (47.2%)	**0.02**
Raised ferritin	3/118 (2.5%)	2/82 (2.4%)	1/36 (2.8%)	1.00
Raised LDH	5/118 (4.2%)	3/82 (3.7%)	2/36 (5.6%)	0.64
Transaminitis	20/118 (16.9%)	11/82 (13.4%)	9/36 (25.0%)	0.18

Age, nationality, presence of comorbidities or concomitant medications did not affect DVS (all p>0.05). Patients isolated in pairs were associated with prolonged DVS (OR: 2.7; 95% CI: 1.7 to 4.5; p<0.0001) compared to those isolated individually. The median (IQR) DVS of patients isolated in pairs was 24 days (23–25), 4 days longer than those isolated individually, with a median of 20 days (17–23). Longer length of symptom duration was associated with prolonged DVS (OR: 1.1; 95% CI: 1.0 to 1.1; p = 0.01) but individual symptoms, type of symptoms, and total number of symptoms were not. Amongst vital signs, only tachycardia was associated with prolonged DVS (OR: 2.6; 95% CI: 1.0 to 7.1; p = 0.03). Amongst blood result abnormalities, only raised CRP was associated with prolonged DVS (OR: 2.7; 95% CI: 1.1 to 6.8; p = 0.02).

Taking day 28 as the cut-off, 187 patients who had a negative swab result up to day 28 (no prolonged DVS) were compared to 126 patients with a persistently positive result on or after day 28 (prolonged DVS) to determine factors affecting DVS.

## Discussion

Young, male patients with few comorbidities, representative of the foreign worker population diagnosed with mild Covid-19 infections in Singapore, were isolated in a community facility. The median duration of viral shedding was 25 days after disease onset and is longer than previously reported in the literature (median 7–19.5 days) amongst patients with non-severe disease [5, 6]. Possibly attributable to the ethnic and gender bias in our population, the discrepancy may also be contributed by the larger proportion of patients in solitary spaces [5] or the administration of empirical antiviral treatment^5^ in other studies.

We found that more patients isolated in pairs were associated with prolonged DVS than those isolated individually, suggesting that high frequency, close contact amongst positive cases increases DVS. Current WHO guidelines recommend that patients in such isolation facilities be placed in single rooms or at a distance of at least one metre apart [7] but we found that patients isolated under these two conditions experience a significant difference in DVS. As the number of infections continue to increase in many countries [8], isolation facilities play an increasingly important role in containment of mildly symptomatic patients without overwhelming the healthcare system. Whether such a perpetuation in viral exposure results in a higher viral load in individual patients and how this translates into viable live virus capable of transmission remains to be investigated. Current evidence suggests that a positive RT-PCR does not equate to detection of live culture and patient infectivity [9]. However, it remains a widely-used and clear-cut surrogate marker for infectivity. Specifically, a negative RT-PCR test result remains an important requirement for air and cruise travel [10] and entrance into certain public places. Further research into the clinical implications of this prolonged DVS is necessary.

In hospitalised patients, corticosteroid administration, presence of fever, and longer time of disease onset to hospitalisation was associated with prolonged DVS [11]. Studies of an older aged population (median age of 81) also found that DVS was significantly longer in the older age group >65 years [12] and >80 years [13], respectively. Similar to previous studies in asymptomatic/pauci-symptomatic populations, our results revealed that being symptomatic or not did not predict DVS [5, 6]. Unlike in our study in which baseline tachycardia and a raised CRP was associated with a prolonged DVS, these were not observed in a previous study [5].

Limitations of the study include the population’s uniform gender and ethnicity which may restrict its external validity. RT-PCR results was reported as binary findings without cycle threshold values and there was a lack of standardised RT-PCR testing schedule protocol although this was likely reflective of real-world practice.

## Conclusion

In summary, in our cohort of young male adults with mild Covid-19 disease, prolonged DVS was strongly associated with being isolated in pairs compared to individually. Tachycardia, and raised CRP were also associated with prolonged DVS but presence or absence of symptoms was not. Our data suggests a risk in using resolution of symptoms as the basis of discharging patients from isolation particularly when DVS can be prolonged in young, mildly symptomatic patients isolated in closed proximity to each other. In countries with sufficient testing capacity and in situations where a negative RT-PCR test result is required, retesting in patients who were not isolated individually, or who had baseline tachycardia or a raised CRP can be postponed to conserve limited laboratory supplies and to increase the yield of a negative result.

## Supporting information

S1 File Minimal underlying dataset. (CSV)
